# First insights into oceanic manta ray movements in Japan using satellite telemetry

**DOI:** 10.1111/jfb.70574

**Published:** 2026-07-19

**Authors:** Rikako Ozaki, Yuko Kamotani, Masato Kamotani, Andrew Chin, Timothy Ravasi, Mark V. Erdmann

**Affiliations:** ^1^ Marine Climate Change Unit Okinawa Institute of Science and Technology Okinawa Japan; ^2^ Japan Manta Project Okinawa Japan; ^3^ Dive Gobies Okinawa Japan; ^4^ Centre for Sustainable Tropical Fisheries and Aquaculture James Cook University Townsville Queensland Australia; ^5^ Faculty of Marine Science and Fisheries Hasanuddin University Makassar Indonesia; ^6^ College of Science and Engineering James Cook University Townsville Queensland Australia; ^7^ Re:wild Austin Texas USA

**Keywords:** *Mobula birostris*, movement ecology, Ryukyu Islands, tagging

## Abstract

Japanese waters represent the northern distributional limit of the western Pacific distribution of the oceanic manta ray (*Mobula birostris)*, yet movement ecology in this region remains poorly understood. We report movements of the first satellite‐tagged individual in Japan. High‐resolution depth–time series data documented repeated dives to 300 m and a maximum depth of 1082 m, with deeper use occurring predominantly at night. The individual undertook repeated long‐distance movements between the Kerama Islands and Miyako Islands, including multiple transits across the Okinawa‐Miyako Submarine Plateau and associated bathymetric features. Although based on a single tracked individual, these observations provide the first high‐resolution movement data of *M. birostris* in Japanese waters and highlight key hypotheses regarding habitat use and connectivity for future investigation.

Japanese waters mark the northernmost distributional limit in the western Pacific for two manta ray species (family Mobulidae): *Mobula alfredi* reef manta ray and *M. birostris* oceanic manta ray (Kashiwagi et al., 2010). Japan also hosts the longest‐running *M. alfredi* photo‐identification database globally, initiated by Takashi Ito in 1977 (Homma et al., 1999; Ishihara & Homma, 1995; Ito, 1987, 2000; Kashiwagi et al., 2011). In addition, the Okinawa Churaumi Aquarium achieved the world's first captive the world's first documented captive birth of *M. alfredi* and has made important contributions to our understanding of their biology and husbandry (Matsumoto & Uchida, 2008; Murakumo et al., 2020).

Based on a systematic assessment of photographic records, Kashiwagi et al. (2010) suggested that a population of approximately 300 *M. alfredi* occur regularly in the Yaeyama Islands (the southernmost islands of Japan) and seasonally in the Miyako and Kerama Islands further north (Figure 1a). In contrast, *M. birostris* were reported regularly only from the Ogasawara Islands, an isolated island group located approximately 970 km southeast of Tokyo (Yano, Ito, et al., 1999) as cited in (Kashiwagi et al., 2010; Yano, Sato, & Takahashi, 1999), with only sporadic records from mainland Japan (i.e., Wakayama, Suruga Bay, Sagami Bay and Aomori; Kawano et al., 2014). More recently, regular occurrences of *M.birostris* have also been documented at Shimozone, a submerged seamount located at the western extent of the Kerama Islands within the Ryukyu Islands (Figure 1a). To our knowledge, Shimozone is currently the only location within the Ryukyu Islands where regular sightings of *M. birostris* have been documented.

**FIGURE 1:**
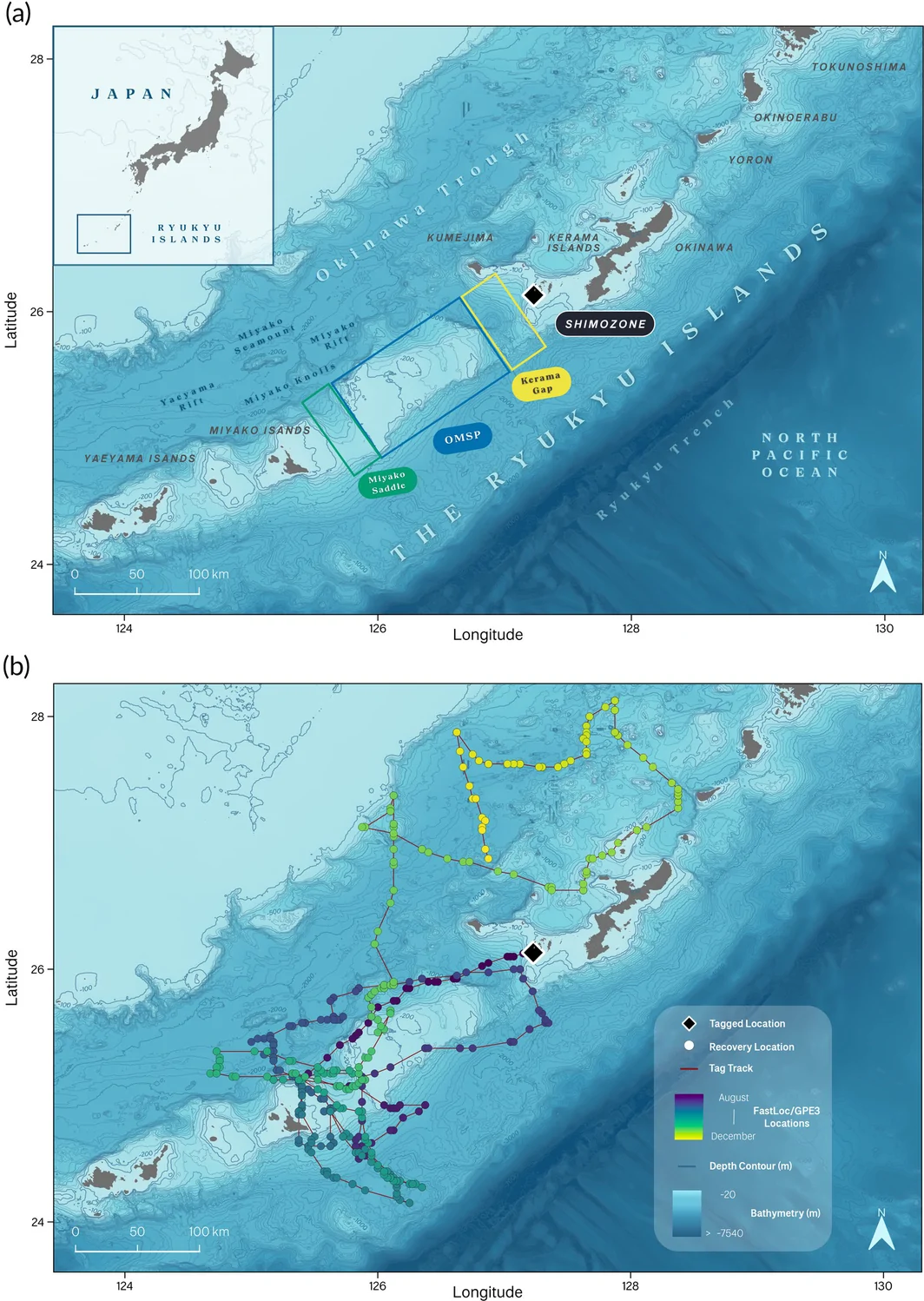
Study region within the Ryukyu Islands. (a) Map showing the tagging location at Shimozone (black diamond) and major bathymetric features, including the Kerama Gap (yellow box), Okinawa‐Miyako Submarine Plateau (OMSP; blue box) and the Miyako Saddle (green box). (b) Estimated movement track of tagged *Mobula birostris* JM‐MB‐0005 from 22 August to 11 December 2024. Colours indicate track progression from deployment (dark blue) to detachment (yellow). The white point indicates the tag recovery location at Hatenohama.

Despite this long history of observations, the spatial ecology and fine‐scale horizontal and vertical movements and habitat use of manta rays in Japanese waters remain poorly understood. This knowledge gap is particularly important given the high mobility of manta rays, their documented occurrence as bycatch in Japanese set‐net fisheries (Kashiwagi et al., 2010; Kimura et al., 2018; R. Ozaki, pers. obs.) and their threatened conservation status. *Mobula alfredi* are listed as Vulnerable and *M. birostris* as Endangered on the IUCN Red List of Threatened Species, following dramatic global population declines (Marshall, Barreto, Carlson, Fernando, Fordham, Francis, Derrick, et al., 2022; Marshall, Barreto, Carlson, Fernando, Fordham, Francis, Herman, et al., 2022). Both species are also listed on Appendices I and II of the Convention on the Conservation of Migratory Species (CMS; Lawson & Fordham, 2018) and have been uplisted from Convention on the International Trade in Endangered Species of Wild Fauna and Flora (CITES) Appendix II to Appendix I, highlighting the conservation concern for these species (Boggio‐Pasqua et al., 2026).

The Ryukyu Islands (Nansei Islands) extend approximately 1200 km from Kyushu, Japan, to Taiwan and comprise a complex system of forearc islands, seamounts, submarine ridges, and offshore bathymetric features, including the Okinawa‐Miyako Submarine Plateau (OMSP), Kerama Gap, and Okinawa Trough (Harigane et al., 2023; Figure 1a). The OMSP is bounded to the northeast by the Kerama Gap, a deep sill separating the Okinawa Islands from the plateau, and to the southwest by the Miyako Saddle, a deep sill separating the OMSP from the Miyako Islands (Nishina et al., 2016; Watanabe et al., 2023). Together, these features form a major bathymetric system extending between the Okinawa and Miyako island groups (Figure 1a).

Shimozone consists of a shallow rocky pinnacle (~8 m depth) that descends steeply before reaching surrounding depths of approximately 200 m (Figure 1a). The site is characterised by strong tidal flow, including both upwelling and downwelling currents, and lies adjacent to deeper bathymetric features associated with the Okinawa Trough and Kerama Gap.

On 22 August 2024, a towed SPLASH10‐F‐321E archival satellite tag (Wildlife Computers, Redmond, WA, USA) was deployed on an adult male *M. birostris* (JR‐MB‐0005) at Shimozone. Disc width was visually estimated at approximately 550 cm while scuba diving alongside the individual during tag deployment. The tag was tethered via a ~ 60 cm stainless steel cable to a titanium dart tip. Deployment was conducted using a modified pole spear or ‘Hawaiian sling’, inserting the dart into the dorsal musculature at the junction between the left pectoral fin and central body cavity to avoid vital organs and minimise interference with swimming, as described in Beale et al. (2025). Following deployment, the individual remained in the area, allowing confirmation of sexual maturity (presence of fully developed claspers) and acquisition of a ventral photograph for photo‐identification.

The tag recorded depth, temperature, and ambient light levels at 1‐s intervals over a programmed 120 day deployment. After detachment, summary histograms of depth and temperature, as well as partial time‐series data at coarser resolution, were transmitted via Argos satellite. Additionally, the tag was physically recovered on 2 February 2025, from Hatenohama, Kumejima. Transmission occurred for 111 days (22 August – 11 December 2024). Recovery of the tag allowed the download of high‐resolution (<15 s) depth and temperature time‐series data, and all analyses were conducted in the R statistical environment (R Core Team, 2025).

Individual dives were extracted from the depth time series using the R package *diveMove* (Luque, 2007). To avoid misclassification of surface behaviours, non‐diving periods were defined as time spent within the upper 50 m of the water column, and dives were defined as periods below this threshold (Beale et al., 2025; Thorrold et al., 2014). Data from the first 24 h following tag deployment were excluded to minimise potential post‐tagging effects, although the individual was visually monitored after deployment and showed no obvious signs of altered behaviour.

SPLASH10‐F‐321E tags are equipped with Fastloc‐GPS receivers, enabling the acquisition of GPS positions during brief surfacing events (~0.2 s; Beale et al., 2025). As these positions only represent intermittent surface locations, maximum‐likelihood movement tracks were additionally estimated using the Wildlife Computers Global Position Estimator 3 (GPE3). GPE3 applies a hidden Markov model to estimate daily positions based on maximum depth, light‐level (twilight) data, sea surface temperature, and known locations (deployment, detachment and resightings; Skomal et al., 2017; Wildlife Computers, 2022). A maximum travel speed of 2.0 m s^−1^ was specified based on the mean speed between high‐quality FastLoc GPS points. Modelled tracks assumed straight‐line movements between estimated positions and did not account for potential land barriers. Consequently, the reconstructed tracks should be interpreted as estimated movement pathways rather than precise movement trajectories, particularly within the complex archipelagic environment of the Ryukyu Islands. High‐quality Fastloc‐GPS positions were used to validate GPE3‐derived tracks where available.

Daytime and nighttime periods were defined using solar times calculated with the R package *suncalc* (Thieurmel & Elmarhraoui, 2025). All timestamps associated with Wildlife Computer archival records and Argos‐derived datasets were converted from Coordinated Universal Time (UTC) to Japan Standard Time (UTC + 9) prior to analysis. Sunrise and sunset times varied by approximately 1 h over the deployment period; therefore, a fixed 12‐h diel division was avoided. Daytime was defined from sunrise plus a 30‐min buffer to sunset plus a 30‐min buffer, with the inverse defined as nighttime, to conservatively account for crepuscular periods and seasonal variation in photoperiod over the deployment period.

To examine patterns in diving behaviour, pairwise comparisons using non‐parametric Mann–Whitney U tests were applied. For this analysis, deep dives were defined as dives deeper than 200 m (Beale et al., 2025; Finucci et al., 2019), and extreme dives as deeper or equal to 500 m (Beale et al., 2025).

The manta exhibited extensive horizontal movements between the Kerama Islands and the Miyako Islands throughout the tracking period (Figure 1b). Following tagging, the manta travelled more than 200 km southwest across the Kerama Gap and OMSP toward the Miyako Saddle within 6 days (Figure 1b), representing the longest straight‐line horizontal displacement recorded during the deployment. The individual subsequently returned northward to the Kerama region (~150 km) before undertaking a second southward movement to the Miyako region later in September, demonstrating repeated long‐distance transits between the two island groups (Figure 1b).

Estimated movement paths revealed a consistent directional pattern along the Kerama Gap and OMSP (Figure 1b). South‐westerly movements from Kerama toward Miyako followed the Kerama Gap and the western margin of the OMSP along the Okinawa Trough, whereas northward movements occurred along the eastern margin (Figure 1b). Mean horizontal displacement over the tracking period was 16.1 km day^−1^.

The manta exhibited pronounced vertical movements throughout the tracking period, reaching a maximum recorded depth of 1082 m. Depth use showed a clear reverse diel vertical migration pattern (Figure 2a), with deeper dives occurring predominantly during nighttime and shallower depth use during the day.

**FIGURE 2:**
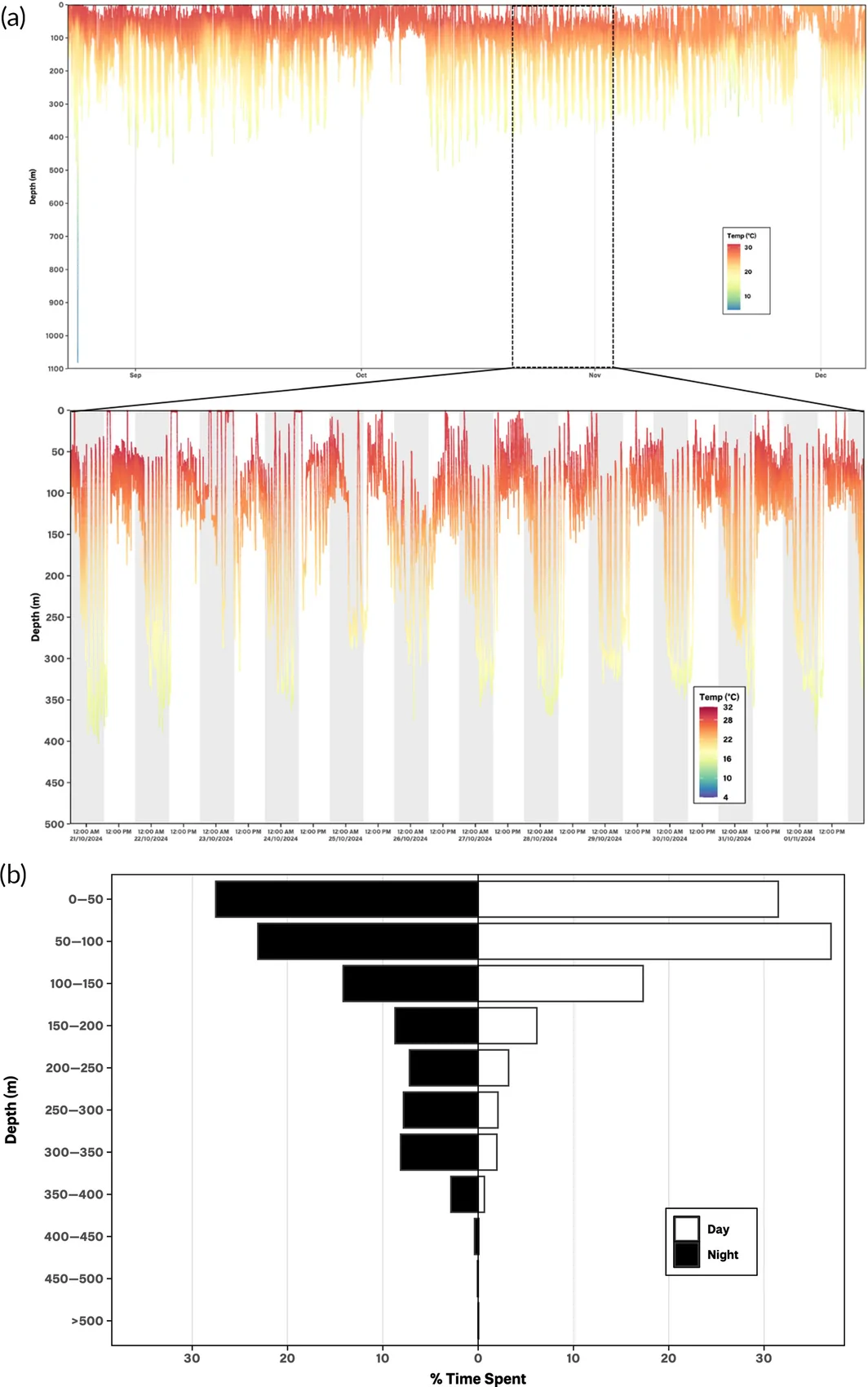
Vertical movement behaviour of *Mobula birostris* JM‐MB‐0005 during the deployment period. (a) Time–depth record over the deployment period, with depth colour‐coded by external temperature (°C). The inset highlights a representative period (21 October to 1 November 2024) illustrating reverse diel vertical migration, characterised by deeper nighttime dives and shallower daytime depth use. (b) Frequency of dive shapes recorded during daytime (white) and nighttime (black) periods.

Across the deployment, the manta moved through a wide thermal range, from 4.4°C at depth to 31.4°C in surface waters (Figure 2a). Mean surface temperature (upper 10 m) declined markedly over the deployment period, decreasing by approximately 4°C from October to November (Supp. Figure 1). Although the proportion of time spent at the surface did not differ significantly among months, surface occupancy tended to increase as surface waters cooled (Supp. Figure 2).

Mean dive depth was greater during nighttime than daytime periods (Mann–Whitney U test, *p* < 0.001; Figure 2b). The manta spent 26.2% of nighttime below 200 m compared with only 7.9% during the day, representing a more than three‐fold increase in deep‐water occupancy.

Dive geometry varied with depth (Figure 3). Shallow dives were dominated by V‐shaped profiles, characterised by brief excursions to depth, whereas mid‐depth and deep dives were predominantly U‐shaped, involving extended bottom phases at maximum depth. The association between dive shape and depth was strong (Cramer's V = 0.416), demonstrating that dive depth exerted a substantial influence on dive geometry. These depth‐related patterns were evident during both daytime and nighttime periods, indicating that dive geometry was more strongly associated with depth than with diel period.

**FIGURE 3:**
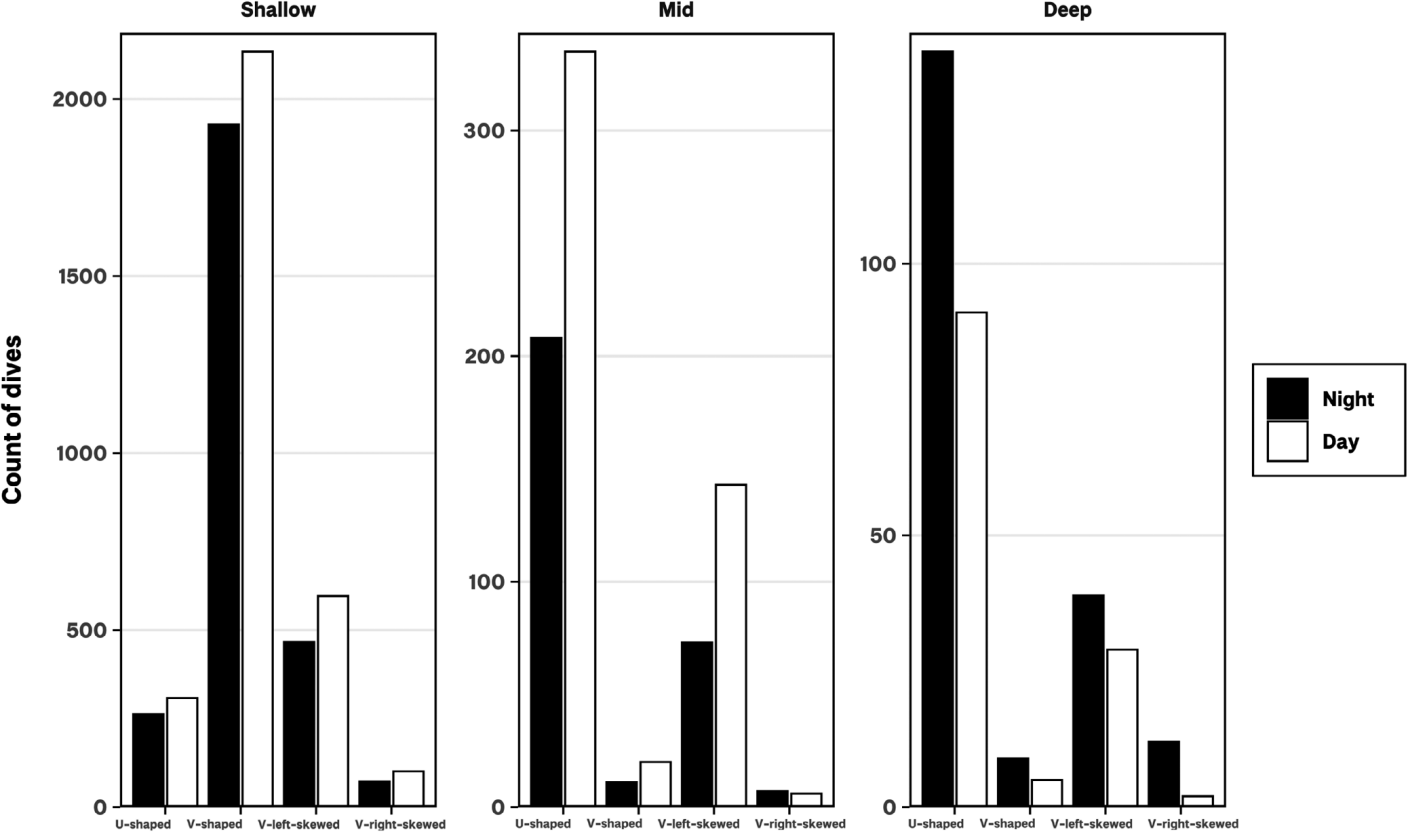
Frequency of dive shapes across depth categories for *Mobula birostris* JR‐MB‐0005. Dives were classified as shallow (<50 m), mid‐depth (50–200 m) and deep (>200 m), and were further separated into daytime and nighttime periods. Bars show the number of dives in each dive‐shape category, with black and white indicating nighttime and daytime dives, respectively. Panels use independent y‐axis scales.

The repeated long‐distance movements observed between the Kerama Islands and the Miyako Islands demonstrate repeated transit between the two island groups by this individual. The apparent use of opposing sides of the OSMP and Kerama Gap depending on travel direction (i.e., western margin during southbound movements and eastern margin during northbound movements) suggests a possible association between movement patterns and regional bathymetric or oceanographic features. However, these records are based on a single tracked individual, further data are required to determine whether these patterns are representative of broader movement behaviour in *M. birostris* within the Ryukyu Islands.

Seamounts and submarine ridges are widely recognised as biological hotspots due to unique oceanographic conditions often associated with upwelling and enhanced prey availability (Morato et al., 2010; Rubin et al., 2025). *Mobula birostris* are frequently observed in association with such offshore features (Kashiwagi et al., 2011; Marshall et al., 2009). Similar associations between *M. birostris* and seamounts or submarine ridges have been reported in other regions, including the Philippines (Barr & Abelson, 2019; Murie et al., 2020), Mexico (Rubin et al., 2025) and the South Atlantic (Beard et al., 2021). The prolonged and repeated occupancy around the Miyako Knolls, Miyako Seamount, Kerama Canyons and Okinawa–Miyako deep‐sea terraces observed in this study is consistent with these previous findings and suggests that offshore bathymetric features likely play an important role in habitat use within the Ryukyu Islands of southern Japan. In this region, interactions between the Kuroshio Current and offshore bathymetric features can generate topographically induced upwelling and enhanced turbulent mixing, increasing nutrient delivery to surface waters despite the generally oligotrophic nature of the Kuroshio Current (Hasegawa et al., 2004, 2008; Nagai et al., 2017; Saito, 2019). Such processes may enhance local productivity around seamounts, ridges, canyons and deep‐sea terraces, potentially increasing prey availability for *M. birostris*.

The pronounced reverse diel vertical migration and greater nighttime occupation of deeper waters observed in this individual are consistent with foraging on vertically migrating mesopelagic prey. Prolonged occupancy at depth and repeated oscillatory diving behaviour during nighttime periods may further suggest active searching or foraging behaviour within deeper water masses. As large planktivores, manta rays are thought to exploit spatially and temporally concentrated prey patches (Meekan et al., 2015), and the observed nighttime diving behaviour may reflect foraging associated with vertically migrating prey within the Okinawa Trough region (Min et al., 2018).

The strong structuring of dive geometry by depth further supports this interpretation. Deeper dives were predominantly U‐shaped profiles and characterised by longer bottom times, a pattern that has been associated with foraging behaviour in previous studies (Beale et al., 2025). In contrast, shallow dives were dominated by V‐shaped profiles with extremely short bottom times, which may reflect behaviours other than sustained foraging, such as environmental sampling or transit. Similar vertical foraging strategies have been proposed for manta rays and other large pelagic fishes, particularly in oligotrophic systems, where access to rising layers of pelagic plankton at nighttime may enhance foraging efficiency (e.g., Braun et al., 2014).

Despite being based on a single individual, this study provides the first high‐resolution vertical and horizontal movement data for manta rays in Japan, at the northern extent of their western Pacific range.

While the movement patterns described here cannot be assumed to be representative of the broader population, the observations provide important preliminary insights into the spatial ecology of *M. birostris* within the Ryukyu Islands and highlight key hypotheses for future investigation. These findings suggest the potential importance of offshore bathymetric features for *M. birostris* in Japan and demonstrate the utility of satellite tagging for resolving movement patterns in regions where traditional monitoring approaches are limited. In particular, the spatial and vertical extent of *M. birostris* movements often lies beyond the effective range of citizen science and other visual‐based methods such as photo‐identification and remote cameras.

While the relationship was not statistically significant, surface occupancy tended to decrease at higher surface temperatures, suggesting a potential influence of temperature on near‐surface habitat use in this individual. However, additional tagging data across multiple individuals and seasons is required to determine whether this pattern is consistent within *M. birostris* in Japanese waters. Expanded tagging efforts are therefore needed to better understand habitat use, movement and connectivity and conservation risks near the northern extent of the species' western Pacific distribution.

## AUTHOR CONTRIBUTIONS

Conceptualisation: Rikako Ozaki, Yuko Kamotani, Masato Kamotani, Mark V. Erdmann. Data curation: Rikako Ozaki, Yuko Kamotani, Masato Kamotani, Mark V. Erdmann. Data analysis: Rikako Ozaki. Funding acquisition: Mark V. Erdmann. Writing – draft: Rikako Ozaki. Writing – revision and editing: Rikako Ozaki, Yuko Kamotani, Masato Kamotani, Andrew Chin, Timothy Ravasi, Mark V. Erdmann. Supervision: Mark V. Erdmann, Andrew Chin, Timothy Ravasi.

## CONFLICT OF INTEREST STATEMENT

The authors declare that the research was conducted in the absence of any commercial or financial relationships that could be construed as a potential conflict of interest.

## Supporting information


**Figure S1.** Monthly mean surface water temperature (upper 10 m) experienced by Maggy (JM‐MB‐0005) during the deployment period (August–December 2024). A significant decline in surface temperature was observed over time (Spearman's rank correlation, *p* = 0.017), the dashed line indicates the fitted linear trend.
**Figure S2.** Relationship between monthly mean surface water temperature (upper 10 m) and the proportion of time Maggy (JM‐MB‐0005) spent in surface waters (<10 m). A negative relationship was observed, with reduced surface occupancy at higher surface temperatures (Spearman's rank correlation, *p* = 0.083). Points represent monthly means; the dashed line indicates the fitted linear trend.

## Data Availability

The data that support the findings of this study are available from the corresponding author upon reasonable request.
