# Supplementary material for: First insights into oceanic manta ray movements in Japan using satellite telemetry
Source: J Fish Biol. 2026 Jul 19;109(4):2052–8. doi: 10.1111/jfb.70574 (PMC13675494; doi:10.1111/jfb.70574)
Supplement: Supplementary file 1 [file JFB-109-2052-s001.docx]

Supplementary Figures

*Supp. Figure 1: Monthly mean surface water temperature (upper 10 m) experienced by Maggy (JM-MB-0005) during the deployment period (August–December 2024). A significant decline in surface temperature was observed over time (Spearman’s rank correlation, p = 0.017), the dashed line indicates the fitted linear trend.*

*Supp. Figure 2: Relationship between monthly mean surface water temperature (upper 10 m) and the proportion of time Maggy (JM-MB-0005) spent in surface waters (<10 m). A negative relationship was observed, with reduced surface occupancy at higher surface temperatures (Spearman’s rank correlation, p = 0.083). Points represent monthly means; the dashed line indicates the fitted linear trend.*
